# Neurorehabilitation robotics: how much control should therapists have?

**DOI:** 10.3389/fnhum.2023.1179418

**Published:** 2023-05-11

**Authors:** Christopher J. Hasson, Julia Manczurowsky, Emily C. Collins, Mathew Yarossi

**Affiliations:** ^1^Department of Physical Therapy, Movement and Rehabilitation Sciences, Northeastern University, Boston, MA, United States; ^2^Department of Bioengineering, Northeastern University, Boston, MA, United States; ^3^Institute for Experiential Robotics, Northeastern University, Boston, MA, United States; ^4^Department of Electrical and Computer Engineering, Northeastern University, Boston, MA, United States

**Keywords:** physical therapy, neurorehabilitation, sensorimotor control and learning, robotics, trust

## Abstract

Robotic technologies for rehabilitating motor impairments from neurological injuries have been the focus of intensive research and capital investment for more than 30 years. However, these devices have failed to convincingly demonstrate greater restoration of patient function compared to conventional therapy. Nevertheless, robots have value in reducing the manual effort required for physical therapists to provide high-intensity, high-dose interventions. In most robotic systems, therapists remain outside the control loop to act as high-level supervisors, selecting and initiating robot control algorithms to achieve a therapeutic goal. The low-level physical interactions between the robot and the patient are handled by adaptive algorithms that can provide progressive therapy. In this perspective, we examine the physical therapist's role in the control of rehabilitation robotics and whether embedding therapists in lower-level robot control loops could enhance rehabilitation outcomes. We discuss how the features of many automated robotic systems, which can provide repeatable patterns of physical interaction, may work against the goal of driving neuroplastic changes that promote retention and generalization of sensorimotor learning in patients. We highlight the benefits and limitations of letting therapists physically interact with patients through online control of robotic rehabilitation systems, and explore the concept of trust in human-robot interaction as it applies to patient-robot-therapist relationships. We conclude by highlighting several open questions to guide the future of therapist-in-the-loop rehabilitation robotics, including how much control to give therapists and possible approaches for having the robotic system learn from therapist-patient interactions.

## 1. Introduction

One of the primary goals of neurorehabilitation is to promote neuroplasticity in sensorimotor networks that generate lasting improvements in patients' functional movement. A key agent of neuroplasticity is a patient's willful activation of neural pathways to produce goal-directed movement (Kaelin-Lang et al., 2005). However, producing voluntary movement with severe neurological impairments is challenging due to various factors such as fatigue, discoordination, or pain. Physical assistance can help minimize these adverse effects, allowing high-repetition training to maximize neuroplasticity and motor recovery.

Physical and occupational therapists are expertly trained to provide manual facilitation, tactile feedback, and verbal coaching to help patients practice functional movements. For example, during gait training, promoting heel strike of the affected limb for an individual who has experienced a stroke can be done by: manually facilitating knee extension during late swing, encouraging dorsiflexor activation by providing tactile feedback to the patient's shin, and providing verbal instruction to extend the knee and strike the heel (Hesse et al., 1994). This active-assisted training can help drive neuroplasticity and motor recovery (Forrester et al., 2008; Mulroy et al., 2010). However, performing these actions over hundreds of steps demands significant therapist physical effort and cognitive attention, which can lead to acute and chronic overuse injuries in therapists (Holder et al., 1999; McCrory et al., 2014).

Increasing the quantity of task-based practice by reducing the physical demands borne by therapists provides a compelling rationale for rehabilitation robots. The expectation is that by providing assisted gait training similar to a therapist, robots can drive neuroplasticity and produce beneficial outcomes. Unfortunately, robotic rehabilitation has, in many cases, not met these expectations. For example, for robotic locomotor rehabilitation, a 2020 Clinical Practice Guideline concluded that current robotic locomotor treadmill training approaches for stroke, spinal cord injury, and brain injury are not recommended (Hornby et al., 2020). There remains a significant need for new avenues of scientific inquiry in robot-mediated rehabilitation.

In this perspective, we focus on the therapist's role in robotic rehabilitation. Early robotic control algorithms did not incorporate human therapist expertise into their design. Although these early robots could produce a large amount of patient movement, the patients often habituated to the robot assistance and decreased their volitional effort (Marchal-Crespo and Reinkensmeyer, 2009; Reinkensmeyer et al., 2009). More recent assist-as-needed algorithms may better mimic therapist approaches to counter habituation by aiming to maximize patient voluntary contributions through active participation (Blank et al., 2014). However, current approaches still do not match the expertise and adaptability of a trained therapist.

With time, as algorithmic advances more closely resemble the strategies employed by human therapists, therapists may find themselves increasingly left out of the robotic control loop. When therapists take on the role of robotic supervisors, removed from direct physical interaction with patients, they lose critical somatosensory information about patient performance and the ability to make on-the-fly adjustments to therapeutic forces applied to patients. We address whether embedding therapists in the lower-level control loops of robotic rehabilitation can enhance rehabilitation outcomes or if instead, we should embrace fully automated robotic systems for clinical care.

## 2. Control of robots—Background

Neurorehabilitation robots are typically programmed to interact with patients autonomously while under clinician oversight; this ensures safety and proper selection of treatment programs. Early approaches for robotic assistance employed position control, using strong motors to move a patient along a predetermined trajectory (Jezernik et al., 2003). While effective at moving the patient, this approach can discourage active patient contributions (Reinkensmeyer et al., 2009; Hornby et al., 2020). In response, impedance-based control systems were developed to increase patients' active participation by modulating robot forces proportionately to volitional movement (Fleerkotte et al., 2014; Meng et al., 2015). Many of these control strategies are designed to assist-as-needed in an attempt to replicate the adaptive training provided by a trained clinician (Cao et al., 2014; Jamwal et al., 2020).

Despite promoting patient involvement, limitations in the ability of robot control algorithms to adapt to changes in patient behavior led to the emergence of a wide variety of human-in-the-loop adaptive control strategies. These algorithms use a combination of sensors to obtain information about human behavior, aided by machine learning, to predict movement intention (Badesa et al., 2014; Khera and Kumar, 2020; Ai et al., 2021; de Miguel-Fernandez et al., 2023). Often, robotic rehabilitation systems require a therapist to tune controller parameters to a patient's needs (de Miguel-Fernandez et al., 2023), but this manual parameter adjustment may occur without critical haptic and kinesthetic feedback about a patient's performance, limiting effectiveness.

Although our aim here is not to exhaustively review robotic control algorithms, we do want to make the point that as time goes on, many robotic assistance strategies seem to more closely mimic what therapists are thought to do, despite there being very few quantitative studies on therapist assistive strategies (Galvez et al., 2005). This begs the question: If an ideal robotic system aims to mimic expert therapists' adaptive training, why not just allow therapists to utilize their clinical expertise via more direct control over patient-robot interactions? We explore several key topics related to this question and the nascent domain of therapist-in-the-loop robotic rehabilitation.

## 3. Control of robots—Therapists in-the-loop

### 3.1. The continuum of control

**Figure 1:**
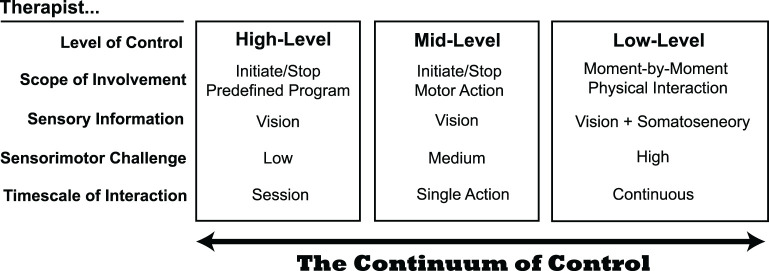
The continuum of control for therapists interacting with a robotic rehabilitation system. At the present time, most systems enable therapist control at mid- to high-levels. Although low-level control presents the largest sensorimotor challenge to therapists, it may provide benefits that the other levels do not, as it permits the most natural physical interaction between a therapist and patient.

We look at therapist-in-the-loop control of rehabilitation robots as a continuum that depends on the timescale of action (Figure 1). Therapist robot control is generally at the highest levels and longest timescales. In the high-level category, therapists are limited to “button-pushers” who initiate, pause, or stop robotic training (Esquenazi et al., 2017). In high-level control, therapists can typically modify robotic routines to adapt to patient needs, for example, if a patient begins to experience pain with a particular movement. The therapist's role is critical in accommodating the non-stationarity of rehabilitation: a patient's physiological status may frequently change, even within a session, causing traditional metrics like averages and variances to lose meaning. However, our limited understanding of how to incorporate therapist feedback may limit the effectiveness of high–level control (Pinheiro et al., 2022).

In contrast to high-level control, there are fewer examples of mid-level control, which operates on a shorter timescale. Instead of monitoring patient progress over a series of exercises, mid-level control allows the therapist to interject on a per-movement basis. Mid-level control is used in some lower extremity exoskeletons, by allowing a therapist to control the timing of when the robot initiates a patient step, for example (Strausser and Kazerooni, 2011; Milia et al., 2016). This intermediate level of control may be useful for patients that have difficulty initiating movements. However, like high-level control, a limitation of mid-level control is that the therapist must rely on their eyes to judge the quality of patient movement; they are unable to use somatosensory feedback as they would if they had their hands on the patient to determine when to alter how the robot is interacting with the patient.

**Figure 2:**
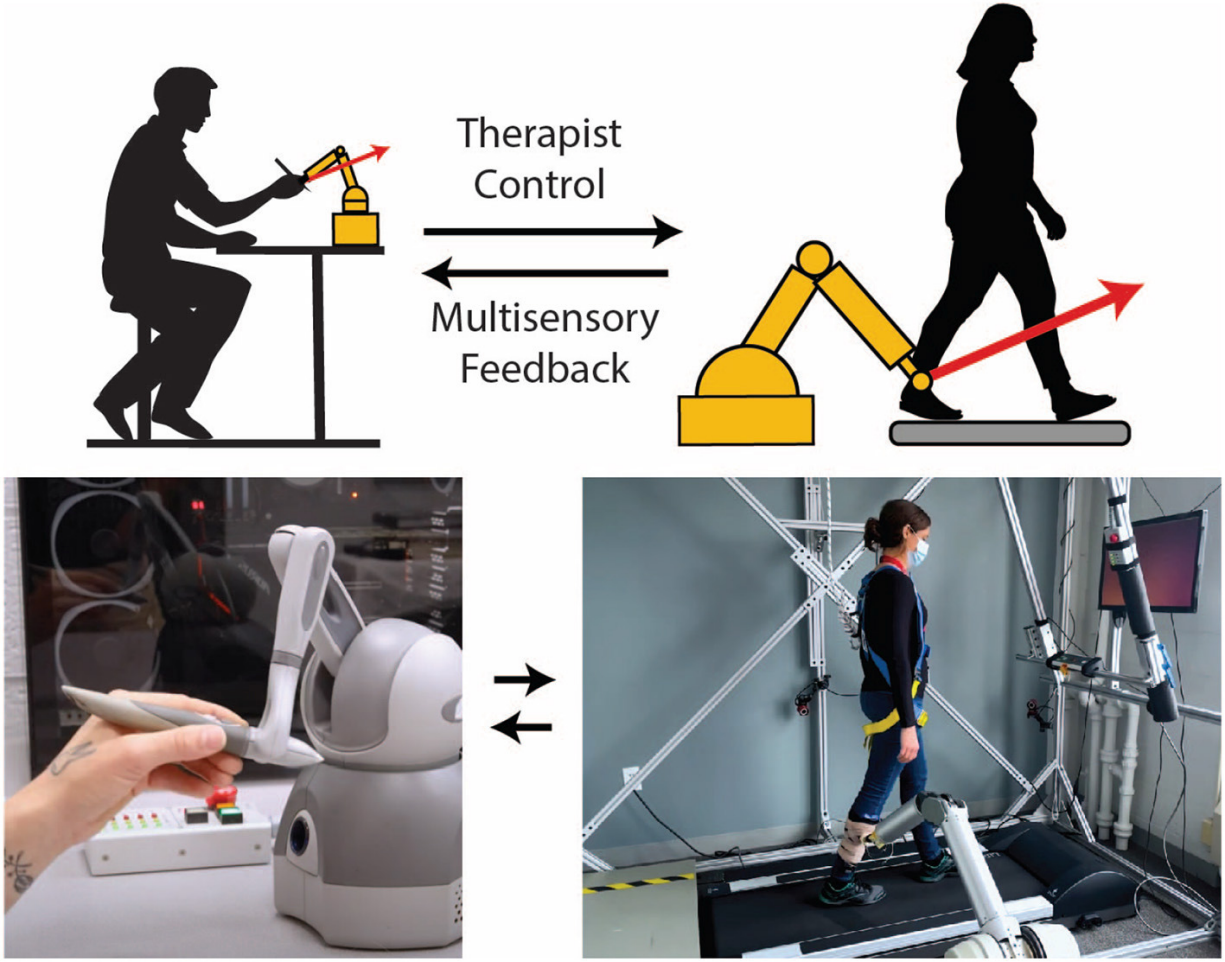
Example of a telerobotic gait training system that gives a physical therapist continuous low-level control over the robotic interaction. In this embodiment, a robotic arm is attached to the patient with a magnetic coupling. Although attached to the leg here, a magnetic attachment facilitates robotic interaction with almost any part of the body if a suitable garment embedded with ferrous material is worn. The motion of the robotic arm is transferred to a small robotic manipulandum held by a therapist (bottom left), so the therapist can feel the patient's motion in near real-time. If the therapist pushes and deflects the manipulandum, the robotic arm will simultaneously apply force to the leg of the patient. This force is amplified and proportional to the therapist's manipulandum force; therefore, the system preserves the time-varying nature of the therapist force, including the natural variability inherent in human interactions.

Low-level therapist control of robotic rehabilitation systems occurs on the shortest timescale. Here, the therapist controls the physical interaction moment-to-moment (e.g., within a single step or a single reaching movement). Only a few examples of low-level control exist today. Teleoperation is one approach for granting therapists low-level control of a robotic rehabilitation system, which allows a therapist to simultaneously feel a patient's movements through haptic feedback and alter how force is applied to the patient through the robotic system (Rahman et al., 2011; Tao et al., 2020). For example, in the system of Koh et al. (2021), the therapist holds onto a small manipulandum that follows the scaled-down movement of a patient's leg during treadmill walking (Figure 2). The operator (or therapist) can assist by deflecting the manipulandum as it follows the patient's motion, and the robot applies a force proportional to the deflection.

For the therapist, low-level robot control is distinguished by a high sensorimotor challenge because the therapist must continuously interact with the complex dynamics of a patient, much like in traditional hands-on facilitation (Hasson and Goodman, 2019). Instead of relying on a computerized controller, the therapist *is* the controller. This could be viewed as a limitation or an advantage, as the effectiveness of therapy becomes dependent on the therapist's skill at controlling the interactive dynamics. Nonetheless, low-level robot control could provide the most human-like interaction for patients, as patients can feel the therapist's actions through the robotic intermediaries. In this way, low-level control preserves the “messiness” of therapist control, which could be advantageous for neurorehabilitation, as discussed next.

### 3.2. Embracing the messiness of human therapists

One of the challenges of robotic rehabilitation is that patients can adapt to assistance in detrimental ways. For example, if the robot pulls the leg upward every step, as might be done to increase step height, a patient may adapt to the presence of this force. Therefore when the assistance force is removed, the patient's step height could become lower (Reinkensmeyer et al., 2006). This phenomenon is explained by research suggesting humans learn internal models (or neural representations) when exposed to novel dynamics (Wolpert et al., 1995). Such models are learned most effectively in deterministic environments, where the relation between forces and motion remains predictable (Bays and Wolpert, 2007).

We propose that the deterministic nature of therapeutic robot control strategies may encourage patients to adapt to a specific algorithm and therefore, improvements may not persist or transfer to other situations and environments. In contrast, therapist assistance is more stochastic and messy. We operationally define messy in terms of the relatively high variability of human motor output caused by sensorimotor noise (Faisal et al., 2008). As a result, a human therapist will never apply the same time-varying assistance force on any two steps (Galvez et al., 2005). This distinctly human characteristic of force delivery could provide a tangible benefit to patients, as variability can be conducive to motor learning and counteract the habituation we previously noted that occurs with deterministic algorithms (Galvez et al., 2011).

Given the potential benefits of a therapist's messy force delivery (compared to a deterministic robotic system), we should consider embracing the stochastic (less predictable) nature of assistance from human therapists. There may also be psychological benefits of this variability, as it may convey the “humanness” of the therapist, and foster patient trust. Without these human qualities, a patient may feel as though they are interacting with an automated robotic system that is emotionless and indifferent to their psychological state and wellbeing. Additionally, these factors may improve patient buy-in to participate in robotic interventions and motivate them to achieve rehabilitation goals. However, the actuality of these benefits remains an open question, as too much messiness and unpredictability could become detrimental to patients.

### 3.3. Therapists teaching machines

The multifariousness of patients may be the most compelling rationale for keeping therapists in the loop, as even the most sophisticated machine learning algorithms can fail when unexpected, contradictory, or complex situations are encountered. Self-driving automobile technology provides a good analogy. For example, on a two-way undivided highway that does not have lane markings, self-driving cars may decide to drive in the center of the lane despite being in the way of oncoming traffic (Linja et al., 2022). Thus, a simple matter for a human driver may become dangerous when left to artificial intelligence to intervene. In our view, the issue is even more complex in robotic rehabilitation.

Unlike self-driving cars, in rehabilitation, the thing being controlled is another living adaptive system, i.e., the patient. In robotic rehabilitation, the controlled entity (the patient) can adapt its behavior drastically from moment to moment in unpredictable ways. Perhaps the best antidote for addressing the unpredictable nature of patient rehabilitation is to use a controller that is equally, if not more, adaptable than the patient (Atashzar et al., 2018). Fortunately, this controller already exists in the form of a human therapist. A therapist is able to adapt how they assist a patient based on patient performance and indicators of a patient's affective state, as well as the level of challenge, fatigue, and pain the patient reports (Sawers and Ting, 2014). At any given time, a therapist may choose to assist, resist, or simply not interact with the patient to promote desired behaviors. The ability of therapists to perform this complex, real-time systems analysis exceeds the current state of the art in adaptive patient-in-the-loop control strategies.

Although human therapists are highly adaptable, continuous low-level robot-mediated physical interaction with patients may place significant attentional and cognitive loads on the therapist. A brief lapse in attention could, for example, result in a resistive, instead of assistive, force being applied to patients. However, a therapist does not need to maintain continuous low-level control if the robot can learn from the therapist. Recently, a learn-and-replay framework has been proposed that includes a therapist-in-loop phase in which the therapist interacts directly with the patient via haptic teleoperation, and a therapist-out-of-loop phase, in which a therapist's previous actions are automated by the robot (Tao et al., 2020). Though sparse in the field of robotic rehabilitation, this type of robotic training represents a form of imitation learning (Schaal, 1999; Fang et al., 2019).

As a final consideration for hybridizing therapist-machine rehabilitation systems, it may be advantageous to envision a system that slides along the continuum of control. For example, machine learning could detect when the algorithmic control of robotic interaction is insufficient, and therapist input is needed. A therapist could also indicate their involvement through their actions. For example, if a therapist is engaged in low-level control by wielding a control interface (e.g., a manipulandum; Figure 2), they could just let go, and the robotic system would take over control. Understanding how aspects of control are shared between the therapist, the patient, and the robot has enormous implications for therapist-in-the-loop systems and, more broadly, the field of rehabilitation robotics.

### 3.4. Who do you trust?

Few would argue that a patient's trust is an essential mediator of therapeutic outcomes. However, how this trust is affected by integrating therapists into robotic control loops is unknown. The specific relationships occurring in human-robot interactions are essential to understand. Exploring trust in social interactions with robots is still a relatively new area of study. Established models of trust in human-robot interaction cover much ground by exploring the human-, robot-, and environmental-related factors influencing trust (Schaefer et al., 2016; Hancock et al., 2021). However, investigating trust toward robots has been focused mainly on cognitive factors, such as beliefs of reliability or capability (Ruff et al., 2002; de Visser and Parasuraman, 2011; Desai et al., 2013). Less is known about how trust is affected when a robotic system is placed between the patient and therapist.

To understand the role of trust in therapist-in-the-loop robotic rehabilitation, we must expand our concept of trust from a *dyad* between the patient and the robot into a *triad* that adds the physical therapist (Cameron and Collins, 2021). In their expert role, physical therapists bring many qualities that impact patient trust. Therapists emphasize building a therapeutic alliance with their patients to create an affective bond and mutual agreement on goals and treatment plans (Kinney et al., 2020). Therapists also constantly evaluate their patients for verbal and nonverbal cues for indications that an alteration is needed. In the parlance of trust in human-robot interaction, the therapist acts as an interested external agent in the therapist-patient-robot triad (Cameron and Collins, 2021), whose actions and relationship with the patient shapes the human-robot interaction. Thus, the therapist is responsible for the robotic system and enabling the patient-robot interaction to occur in the form it does.

One might expect that a patient's trust in rehabilitation robots will increase with the therapist-in-the-loop. However, reality might not be so straight forward. For example, even if the robot malfunctions by no fault of the therapist, the experience may destroy the trust the patient has for the therapist. Thus, work in this area of trust and rehabilitation robotics would allow us to better pick which kinds of human-in-the-loop systems to design. By learning how trust impacts the continuum of control, it will become clearer how the robot is trusted as an extension of the therapist, and the influence trust has on robotic rehabilitation outcomes.

## 4. Discussion

In this perspective, we explored how embedding therapists in the lower-level control loops of robotic rehabilitation systems may improve neuroplasticity and rehabilitation outcomes. The tight integration of therapists in robotic control embraces the variability of human therapists, leverages their vast clinical experience, and maintains a distinctly human link with patients that could enhance patients' trust in robotic technology. Below, we pose a set of forward-looking questions for which answers will take an interdisciplinary investigative approach from multiple stakeholders, including patients, therapists, neuroscientists, and engineers:

***Questions for patients:***

- How is patient perception mediated by knowledge of whether the robot is under therapist or automated control?
- Does giving therapists more control of robotic interactions inspire greater patient engagement in task-based practice and better help them achieve their rehabilitation goals?
- How does the level of therapist control affect patient trust in using robotics for rehabilitation?

***Questions for physical therapists:***

- How much control over physically-assisted robotic interventions do therapists want?
- What training is needed for therapists to safely manage low-level physical interactions using a robotic rehabilitation system?
- How can therapists maintain trust and a solid therapeutic alliance with patients using robotic systems?

***Questions for neuroscientists:***

- What aspects of traditional hands-on therapy (variability, adaptability, etc.) are critical to promoting neuroplasticity and behavioral modification with robotic systems?
- How much control do therapists need to maximize their effectiveness at promoting neuroplasticity?
- What kind of feedback about the robotic system and patient performance is optimal to guide therapist intervention?

***Questions for engineers:***

- How should the therapist interface be constructed for optimal transmission of therapist assistance?
- Can algorithms be designed so the robotic system can learn from therapist trainers (and vice-versa)?
- How can a robotic system be programmed to mimic the variability inherent in therapist-provided assistance and feedback?

***A question for all:***

- How can each stakeholder, i.e., therapists, neuroscientists, and engineers, best incorporate feedback from other stakeholders to optimize rehabilitation outcomes for patients?

## Data availability statement

The original contributions presented in the study are included in the article/supplementary material, further inquiries can be directed to the corresponding author.

## Author contributions

CH drafted the manuscript. CH, JM, EC, and MY edited the manuscript and made significant intellectual contributions. All authors contributed to the article and approved the submitted version.
